# Platelet-Rich Plasma Supplemented Revascularization of an Immature Tooth Associated with a Periapical Lesion in a 40-Year-Old Man

**DOI:** 10.1155/2014/479584

**Published:** 2014-02-20

**Authors:** Ganesh Ranganath Jadhav, Naseem Shah, Ajay Logani

**Affiliations:** Department of Conservative Dentistry and Endodontics, Centre for Dental Education and Research, All India Institute of Medical Sciences, New Delhi 110029, India

## Abstract

The present case report is the first of its kind that documents the successful outcome of “revascularization,” a regeneration-based treatment protocol in a mature adult patient. It belies the myth that “revascularization” should only be done in children and young, adolescent patients. The misconception that stem cells number as well as viability in older age group patients will not allow revascularization to be successful is also contradicted by this case. The paper highlights all the mechanisms that come into play and the enhancing of regenerative response by supplementation with platelet-rich plasma (PRP).

## 1. Introduction

Revascularization as a treatment option for the management of nonvital, immature teeth is an established ADA procedure code [1]. Numerous case reports/case series have documented its efficacy and success. Till date, its application has been limited to the patients between the age group of 8 and 16 years [2] and in teeth with minimal periapical pathology. However the scope of this procedure stands to change with the growing evidence of persistence of self-maintained mesenchymal stem cells (MSCs) in adults [3] and advent of superior scaffolds like platelet-rich plasma and platelet-rich fibrin that delivers enhanced concoction of growth factors. In addition, the presence of inflamed periapical progenitor cells (iPAPCs) [4], infection survived stem cells from apical papilla (SCAP) [5] in teeth with large periapical pathology, may further widen its horizon. The present case reports the use of platelet-rich plasma as a supplementary scaffold for successful revascularization of a maxillary lateral incisor associated with large periapical lesion in a forty-year-old Asian male.

## 2. Case Report

**Figure 1:**
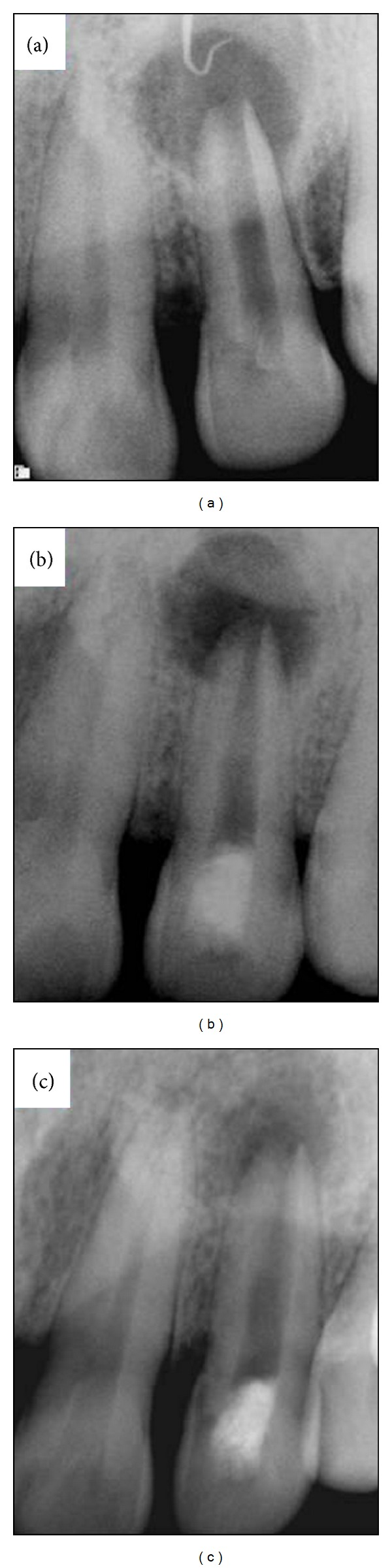
(a) An immature, maxillary right lateral incisor with open apex and large localized, well-defined periapical radiolucency in a 40-year-old male. (b) Six months after revascularization + PRP, apical pathology is reduced and apical narrowing is seen. (c) At 1-year follow-up, normal bony architecture around the entire root is reestablished. Note the narrowing of the apical third of the root canal.

A 40-year-old healthy Asian male presented with the chief complaint of intermittent pus discharge from the right upper gums. Patient gave a history of trauma sustained thirty years ago while playing. Clinical examination revealed a discolored right maxillary lateral incisor and an associated intraoral draining sinus in the labial vestibular region. On palpation a localized bony hard swelling was discernible. The tooth was nontender to percussion and had a grade I mobility. Vitality tests (cold and electric pulp tests) were negative. Intraoral tracer periapical radiograph with a #30 gutta-percha point was exposed (Figure 1(a)). It revealed a blunderbuss root apex with thin lateral dentinal walls associated with a large periapical pathology measuring 1.5 × 1.5 cm^2^ (Figure 1(a)). Based on clinical, pulp sensibility and radiographic findings, a diagnosis of pulp necrosis with chronic apical periodontitis in relation to #22 was established. The patient was explained regarding the treatment options. Taking into consideration the wide-open apex, stage of root development (Nolla's ninth stage of root development), and maturation of the dentinal walls, revascularization with platelet-rich plasma was preferred. The risks, complications, and possible outcome of this treatment were explained and patient's written informed consent was obtained.

Endodontic access cavity was prepared using appropriate armamentarium (LA Axxess kit) (Sybronendo, CA, USA) under rubber dam isolation. The canal was copiously flushed with triple distilled water. Electronic working length (Root ZX; Morita, Tokyo, Japan) was determined. Using this length, ISO #80 K-file was introduced into the canal and a radiograph was exposed. The final working length was established by placing a corresponding sized paper point to the predetermined length and advanced till a dry paper point was achievable. Minimal mechanical instrumentation with ISO #80 H-file (Dentsply Maillefer, Tulsa, OK) and copious irrigation with 20 mL of 2.5% sodium hypochlorite (NaOCl, Cmident (India)) was performed. The canal was dried. Interappointment medication of triple antibiotic paste [6] was applied. Tooth was temporarily restored with Intermediate Restorative Material (IRM, Caulk DENTSPLY, Milford, DE).

The patient was recalled after three weeks. Subsequent to resolution of clinical signs and symptoms, the tooth was reaccessed. Local anesthesia without adrenaline (LOX 2% Neon Lab, India) was deposited in the vicinity of the apex of #22. A final rinse with 17% EDTA was done. Revascularization was performed. A sterile #25 K-file with a rubber stopper set at 2 mm beyond the established working length was pushed past the confines of the canal into the periapical tissue to induce bleeding. Cotton was held in the pulp chamber for 5–7 minutes to allow blood clot formation in apical one third of the canal. For chair side preparation of PRP, 8 mL of blood drawn by venipuncture of the antecubital vein was collected in a 10 mL sterile glass tube coated with an anticoagulant (acid citrate dextrose). This was centrifuged at 2,400 rpm for 10 minutes to separate PRP and platelet-poor plasma (PPP) from the red blood cell fraction. Supernatant layer (PRP + PPP) was transferred to another tube and again centrifuged at 3,600 rpm for 15 minutes to separate the PRP from the PPP. At the end of this cycle, PRP precipitated at the bottom of the glass tube. This was retrieved with sterile cotton pliers and placed on a glass petridish. It was mixed with 1 mL of 10% calcium chloride to activate the platelets and to neutralize the acidity of acid citrate dextrose. PRP procured by above mentioned procedure was then introduced into the pulp chamber with the help of sterile cotton pliers and carried to the apical portion with a size 40 finger plugger (Sybronendo, CA, USA). Access opening was sealed with resin modified glass ionomer cement (Photac-Fill, 3MESPE, Minnesota). Baseline and subsequent 6- and 12-month follow-up intraoral radiographs were taken. The patient was clinically asymptomatic with complete resolution of intraoral sinus and swelling. Periapical healing, apical closure, root lengthening, and dentinal wall thickening were evident radiographically (Figure 1(c)).

## 3. Discussion

Revascularization is now recognized as an accepted treatment protocol for management of immature, nonvital, infected teeth. In the past over a decade, several case reports, case series, and review articles have established their efficacy in management of immature, infected teeth. In the past decade, this protocol has undergone many evolutions to enhance the regenerative response to this novel technique of strengthening an immature tooth by deposition of mineralized tissues and revitalizing the nonvital tooth.

Irrigation protocols and intracanal dressings have the potential to affect stem cell survival adjacent to the walls of the root canal system and those residing in the periapical tissues, possibly by direct and indirect mechanisms [7]. The irrigation regime has varied from 3% hydrogen peroxide [8], 2% chlorhexidine to 2.5–6% NaOCl. However, chlorhexidine was found to be detrimental to the SCAP [7]. Use of 17% EDTA is reported to promote SCAP survival and attachment to the dentinal walls of the root canal and aids in the release of growth factors from demineralized dentin. Hence EDTA is recommended as a final rinse in cases of revascularization [7]. Intracanal dressing of calcium hydroxide has been traditionally employed in the treatment of immature teeth [4]. But its role in revascularization is being debated. Its high pH is believed to reduce the viability and regenerative capacity of the apical vital cells [9]. But the other view is that calcium hydroxide stimulates the Hertwig's epithelial root sheath and hence it is useful in developing proper root morphology [10]. Current consensus about the placement of calcium hydroxide is that it should be restricted to the coronal half of the root canal system for its potent antibacterial property for effective canal disinfection and, at the same time, avoid any detrimental effect on viability of cells in the periapical region [4]. Use of triple antibiotic paste consisting of ciprofloxacin, metronidazole, and minocycline was proposed by Hoshino et al. for improved disinfection in cases of revascularization [6, 11]. But its use can cause crown discoloration, which is attributed to minocycline. To overcome this limitation, minocycline is either removed from the antibiotic paste (double antibiotic paste) or substituted with cefaclor [12]. Cohenca et al. suggested the use of the apical negative pressure irrigation system, that is, EndoVac in lieu of intracanal antibiotics for effective disinfection in revascularization [13].

Initially revascularization procedure involved overinstrumentation beyond the confines of the canal into the peri-apex to induce a blood clot formation. This acted as a natural scaffold [14]. However the concentration of growth factors is limited and unpredictable. Furthermore, erythrocytes in the clot undergo necrosis, affecting its properties [15]. Thibodeau et al. incorporated collagen as a scaffold for the revascularization procedure in dogs. From their results, no statistically significant evidence supporting the promotion of revascularization by a collagen solution scaffold was found [16]. The authors conducted a pilot clinical study to evaluate revascularization with and without platelet-rich plasma in nonvital, immature, anterior teeth. Study concluded that supplementations with PRP can potentially improve the desired biological outcome of this regenerative technique [17]. Shivashankar et al. have reported a case of revitalization with platelet-rich fibrin (PRF) as a scaffold [18]. They concluded that PRF is an ideal biomaterial for pulp-dentin complex regeneration. A double seal of MTA and resin-modified glass ionomer cement is recommended. Ideally, the coronal edge of MTA should be placed 1 to 2 mm apical to the CEJ versus 3 to 4 mm as recommended by Banchs and Trope to allow for more root development [19]. Nosrat et al. suggested the use of calcium enriched mixture (CEM) in place of MTA [20]. CEM might promote the process of differentiation of stem cells and induce the formation of dentin-like hard tissue.

Till date, revascularization protocol has been limited to adolescent patients between the age group of 8 and 16 years. There is a perceived notion that a decrease in regenerative potential of stem cells with ageing can occur, in quantity, quality (differentiation/regeneration capacity), and mobilization capacity. However there is growing evidence that the MSCs recruited in the regenerative endodontic procedure are either less prone to aging (‘‘persisting MSCs”) or elude aging indefinitely (‘‘perennial MSCs”-Bone Marrow Mesenchymal Stem Cells (BMMSCs)) [21]. In addition, Song et al. documented *in vivo* that nonproliferative, quiescent adult MSCs can be stimulated by the signals triggered either by tissue damage or by different growth factors released from the scaffold [22].

The existence of stem cells in teeth with a large periapical pathosis is uncertain. However, there is a growing body of evidence that stem cells like inflamed periapical progenitor cells (iPAPCs) and stem cells from apical area (SCAP) can exist in nonvital teeth with apical periodontitis. Under the organizing influence of infection resistant Hertwig's epithelial root sheath (HERS), these cells can favorably induce maturogenesis in such teeth. In revascularization, overinstrumentation into the periapical region triggers an inflow of blood into the root canal space and also releases growth factors by causing trauma. The blood clot has been shown to have a 400–600 fold greater concentration of mesenchymal stem cell markers (CD73 and CD105) as compared to their concentration in the circulating systemic blood [23]. Supplementation with PRP has been shown to improve the outcome of revascularization [17]. Several growth factors, such as transforming growth factor-1 and vascular endothelial growth factors, released from the platelets stimulate stem cells and enhance their regenerative potential. In addition, it stimulates collagen synthesis, controls local inflammation, and thereby improves tissue healing.

In the present case, proper disinfection, induction of intentional tissue inflammation and use of a suitable scaffold (blood clot + PRP) led to successful outcome of revascularization in the adult patient. With better understanding regarding the survival and behavior of the MSCs, the application of this regenerative procedure to adult teeth was logical and was based on sound, available scientific literature. However, for it to be established as an evidence-based treatment protocol for adult patients, further long term clinical studies with standardized protocols and objective assessment of its efficacy by 3D imaging need to be conducted.
